# Long-acting growth hormone in the treatment of growth hormone deficiency in children: a systematic literature review and network meta-analysis

**DOI:** 10.1038/s41598-024-58616-4

**Published:** 2024-04-05

**Authors:** Jianfang Zhu, Ke Yuan, Sunita Rana, Satya Lavanya Jakki, Amit Subray Bhat, Li Liang, Chunlin Wang

**Affiliations:** 1 Department of Pediatrics, The First Affiliated Hospital, Zhejiang University School of Medicine, Hangzhou, 310003 Zhejiang Province China; 2 Indegene Limited, Bengaluru, 560 045 India

**Keywords:** Growth hormone, Growth hormone deficiency, Height velocity, Long-acting growth hormones, Short stature, Endocrinology, Health care

## Abstract

The purpose of this study is to compare the relative efficacy and safety of long-acting growth hormone (LAGH) as a growth hormone replacement therapy in prepubertal children with growth hormone deficiency (GHD). We searched the PubMed, Embase, CNKI, and Wanfang databases from inception to July 2023 and identified eleven relevant studies. PEG-LAGH showed better effect on height velocity (mean difference [MD]: − 0.031, 95% credibility interval [CrI]: − 0.278, 0.215) than somatrogon (MD: 0.105, 95% CrI: − 0.419, 0.636), somapacitan (MD: 0.802, 95% CrI: − 0.451, 2.068) and lonapegsomatropin (MD: 1.335, 95% CrI: − 0.3, 2.989) when compared with daily growth hormone (DGH). Furthermore, in terms of height standard deviation score, PEG-LAGH demonstrated better improvement (MD: − 0.15, 95% CrI: − 1.1, 0.66) than somatrogon (MD: − 0.055, 95% CrI: − 1.3, 0.51) and somapacitan (MD: 0.22, 95% CrI: − 0.91, 1.3). PEG-LAGH (risk ratio [RR]: 1.00, 95% CrI: 0.82, 1.2) reduced the risk of adverse events compared with other LAGH (somatrogon, RR: 1.1, 95% CrI: 0.98, 1.2; somapacitan, RR: 1.1, 95% CrI: 0.96, 1.4; lonapegsomatropin, RR, 1.1, 95% CrI: 0.91, 1.3) and was comparable with DGH. This is the first study to indirectly compare the LAGH thorough a network meta-analysis and provide evidence of the optimal efficacy of various LAGH specifically PEG-LAGH and acceptable safety profile in prepubertal children with GHD.

## Introduction

Children with growth hormone deficiency (GHD) is characterized by diminished growth velocity and abnormal linear growth resulting in a short stature^1^. The consensus guidelines of the ‘Growth Hormone Research Society’ recommends the use of recombinant human growth hormone (rhGH) for the treatment of GHD in childhood and adolescence stage^2^. To achieve a primary goal of growth promotion and normalize the final adult height, treatment with rhGH has been well-established for patients with GHD for decades^3,4^. Multiple clinical trials have demonstrated the efficacy and safety of rhGH therapy^5–8^. However, despite its long-proven efficacy and safety, conventional rhGH replacement therapy requires daily injection resulting in poor compliance thereby, impacting the treatment outcome^9,10^. A systematic literature review reported 71% of patients with GHD to be non-adherent to the prescribed treatment^11^.

To reduce the frequency of administration and improve adherence to therapy, various strategies have been explored. One such strategy is the long-acting GH (LAGH) formulations with longer dosing intervals to mitigate non-compliance associated with daily growth hormone (DGH). Innumerable LAGH formulations have been developed, each with unique molecular characteristics. Sogroya® (somapacitan), a once-weekly LAGH has been approved for the treatment of adults and children in Europe^12^ and the United States Food and Drug Administration (US FDA)^13^, whereas approved only for adults in Japan^14^. Another LAGH, Skytrofa® (lonapegsomatropin, TransCon hGH), has also been approved by the US FDA and Europe for the treatment of pediatric GHD^15^. NGENLA® (somatrogon) is a long-acting rhGH currently approved in United States, Canada, Australia, Japan, the United Kingdom, and the European Union as a once-weekly subcutaneous injection for the treatment of pediatric GHD^16^. Jintrolong®, a polyethylene glycol LAGH (PEG-LAGH) has been approved in China for children with GHD. The clinical testing of PEG-LAGH started with the human tolerability trial and single-dose PK trial on healthy volunteers^17^, followed by phase 2 and 3 trials involving six Chinese hospitals^18^.

Multiple studies have demonstrated comparable beneficial efficacy of LAGH with DGH for the treatment of GHD with no additional incidence of adverse events (AEs)^19–21^. Till date, the LAGH analogs, have not shown substantial differences in terms of efficacy and safety compared to DGH, except for PEG-LAGH which demonstrated a significant better efficacy as compared with DGH especially at lower doses^18^. Despite this promising efficacy, the results should be interpreted with caution as the activity was up to 25 weeks only and further long-term studies are needed to conclude the effects. Recently, an increased incidence of cardiovascular and cerebrovascular mortality after long-term treatment with daily rhGH was reported^22^. Thus, further evaluation of the effective LAGH is necessary to treat patients with GHD. Of the various LAGH, somapacitan, lonapegsomatropin, somatrogon and PEG-LAGH have established their clinical efficacies in several clinical trials whereas other LAGH preparations faced major setbacks and challenges^14,23^. Besides, it has been observed that these four drug molecules exhibit similar efficacies. With this rationale, the above mentioned LAGH were selected for this current study to identify the optimal treatment for prepubertal children with GHD. So far, there has been no head-to head comparison of LAGH in children with GHD. Hence, the present network meta-analysis (NMA) study aims to indirectly investigate the relative efficacy and safety of various LAGH for the treatment of prepubertal children with GHD.

## Methods

This NMA followed the “Preferred Reporting Items for Systematic reviews and Meta-Analyses (PRISMA)” guidelines^24^ and was registered in the International Prospective Register of Systematic Reviews (PROSPERO registration number: CRD4202339043).

### Data sources and search strategy

An extensive search was carried out for articles published from inception to July 2023 in databases such as PubMed and Embase to identify relevant English articles using following search strings with minor changes in Boolean signs to suit the database: “growth hormone deficiency”, “jintrolong”, “somapacitan”, “Somatrogon”,. “lonapegsomatropin”, “rhGH-PEG”, “long-acting growth hormone”, “daily growth hormone”, “short-acting growth hormone”. Similarly, search was carried out in Chinese databases CNKI and Wanfang (Supplementary Table 1). To ensure completeness, various commentaries, editorials, and conference publications were hand-searched.

### Study selection

Studies were included if they met the following criteria: 1) randomized controlled trial (RCT) 2) growth hormone deficiency in prepubertal children; 3) receiving PEG-LAGH, somapacitan, somatrogon and lonapegsomatropin. Case reports, post-hoc analysis, reviews, meta-analysis, cost related studies, studies published in language other than in English and Chinese, and studies on non-human research subjects were excluded.

### Data extraction and quality assessment

Data were extracted based on aforementioned inclusion and exclusion criteria (e.g., study ID, sample size, patient age, and gender), treatments (interventional and controlled arms) as well as outcome in a spreadsheet. To avoid any potential assessment bias, two independent reviewers selected the studies and performed data extraction. Any discrepancies during the extraction were resolved by discussion. Jadad score was used to score the included studies and assess the risk of bias^25^ based on: randomization (0 to 2 scores), blinding (0 to 2 scores), and dropouts and withdrawals (0 to 1 score). All the relevant articles were given a score of 4 to 5 (high quality), 3 (medium quality) and 0 to 2 (low quality)^26^. Publication bias was evaluated by Begg’s rank test and Egger’s test for all the efficacy and safety outcomes.

### Data synthesis and statistical analysis

Bayesian approach was used for the relative evidence, such as mean difference ([MD], (95% credible intervals [95% CrI]) for efficacy outcomes and risk ratio (RR) for adverse events (AEs). The primary endpoint of the study was height velocity (HV) and height standard deviation score (HSDS) whereas safety based on AEs was the secondary endpoint. NMA was performed by using “Gemtc” 4.0.4 package to run Bayesian analyses (R Foundation for Statistical Computing, Vienna, Austria). The statistical inference in this study was based on credible intervals or confidence intervals rather than *p*-values. These intervals provide a range of values within which the true effect is likely to lie with a certain level of confidence. Clinical heterogeneity was assessed using the inconsistency index (I^2^-statistic) that describes the percentage of total variation across studies that is due to heterogeneity rather than chance. Random effects model was used for high level of heterogeneity (> 60%) and fixed effects model was used for low (≤ 30%) and moderate (≤ 60%) levels of heterogeneity. To rank the intervention, surface under the cumulative ranking curve (SUCRA) was calculated. As the SUCRA value became larger, the treatment demonstrated better efficacy^27^. A *p* value < 0.01 indicates publication bias and in case of publication bias, the study was removed and the outcomes were rechecked.

## Results

### Selection of eligible articles

**Figure 1:**
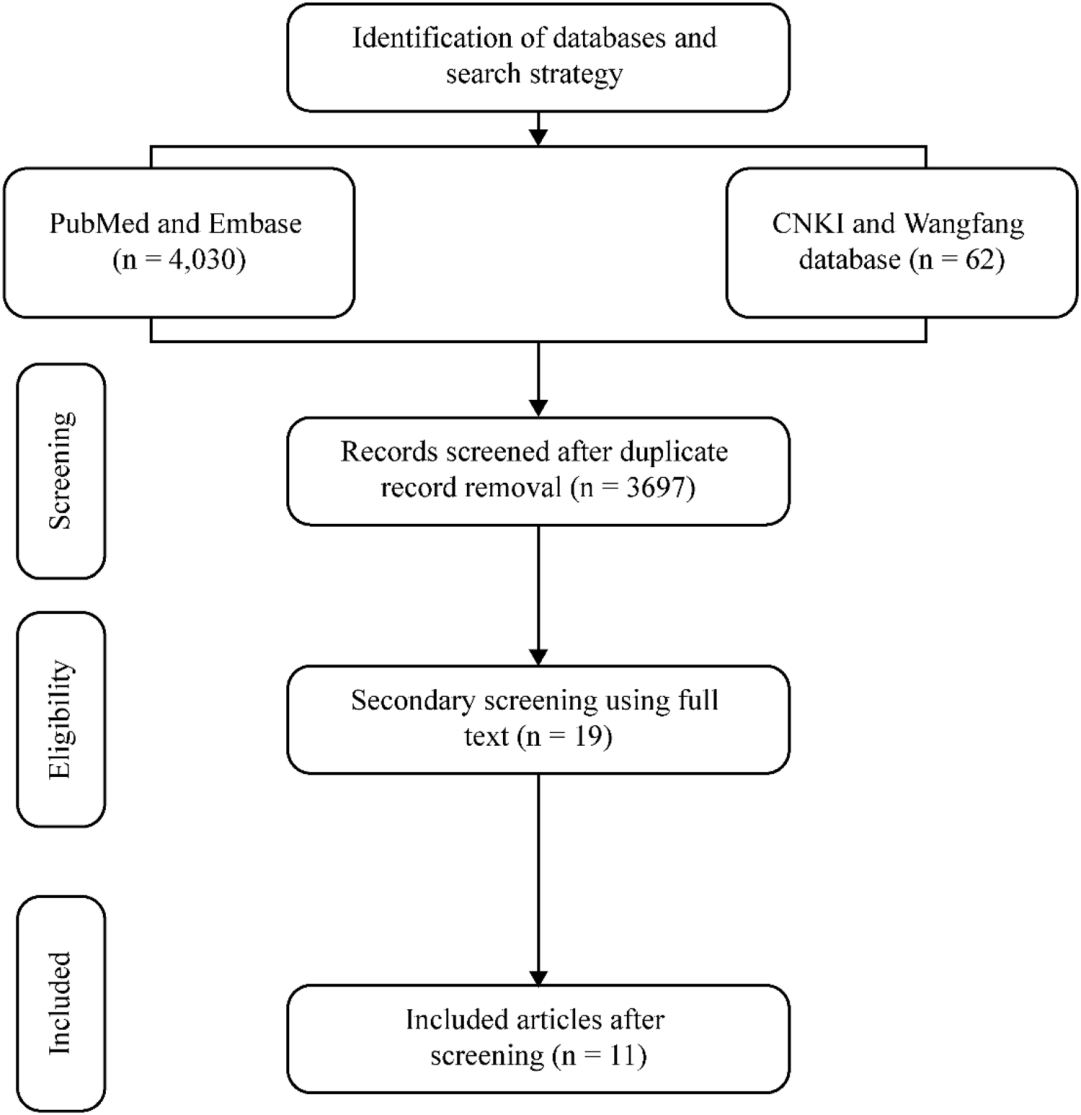
PRISMA flow chart.

The initial electronic search yielded 4,092 consolidated studies from the selected databases and 3,697 articles remained after removing the duplicates. Articles were further screened based on the title and abstract to determine eligibility. Upon full text screening of these articles, 11^16,18,22,28–35^ RCTs were considered eligible for final analysis. Figure 1 represents the study flow diagram as per PRISMA guidelines.

### Study characteristics

One Chinese and ten English articles were included in the final analysis. A total of 1,899 patients were included in the present analysis, of which 1,222 were in the LAGH group and 677 were in the DGH group. All study results were published between 2017 to 2022. The study characteristics and the interventions in the included studies are provided in Table 1. Quality assessment of the RCTs by JADAD score revealed that most of the studies were of low quality. The details of risk of bias assessment in each domain are presented in Table 1.

**Table 1:** Study characteristics.

Author and year	NCT number	Study design	Intervention (I)		Comparator (C)		Patients randomized (N)	Treatment duration	JADAD Score
			Drug	Dose	Drug	Dose			
Miller et al.^35^	NCT03811535	Randomised, multinational, open-labeled, active-controlled parallel group phase 3 trial	Somapacitan	0.16 mg/kg/wk	Daily GH	0.24 mg/kg/wk	I: 132 C: 68	52 weeks	2
Sävendahl et al. ^22^	NCT02616562	Multicenter, randomized, controlled, phase 2 study	Somapacitan	0.04 mg/kg/wk; 0.08 mg/kg/wk; 0.16 mg/kg/wk	Daily GH	0.24 mg/kg/wk	I: 43 C: 14	52 weeks	4
Du et al.^29^	–	Single-center, open-label, prospective, randomized controlled trial	PEG-LAGH	0.12 mg/kg/wk; 0.20 mg/kg/wk	Daily GH	0.28 mg/kg/wk	I: 48 C: 23	52 weeks	2
Sun et al.^30^	NCT02976675	Phase IV, randomized, open-label, parallel-group, noninferiority trial	PEG-LAGH	0.20 mg/kg/wk	Daily GH	0.25 mg/kg/wk	I: 372 C: 176	26 weeks	3
Thornton et al.^28^	NCT02781727	Randomized, open-label, active-controlled, Phase 3 trial	Lonapegsomatropin	0.24 mg/kg/wk	Daily GH	0.24 mg /kg/wk	I: 105 C: 56	52 weeks	2
Luo et al.^18^	NCT01342146, NCT01495468	Multicenter, randomized, open-label, phase 2 and 3 trials	PEG-LAGH	0.1 mg/kg; 0.20 mg/kg/wk	Daily GH	0.25 mg/kg/wk	I: 61 (phase 2), 228 (phase 3) C: 34 (phase 2), 115 (phase 3)	25 weeks	2
Chatelain et al.^31^	NCT01947907	Multicenter, Phase 2, Randomized, Open Label, Study	Lonapegsomatropin	0.14, 0.21, 0.30 mg/kg/wk	Daily GH	0.21 mg/kg/wk	I: 40 C: 13	26 weeks	2
Wan et al.^32^	–	Randomized controlled trial	PEG-LAGH	0.20 mg/kg/wk	Daily GH	0.03 mg/kg/wk	I: 20 C: 30	52 weeks	-
Deal et al.^16^	NCT02968004	Open-label, multicenter, randomized, active-controlled, parallel-group, phase 3 study	Somatrogon	0.66 mg/kg/wk	Daily GH	0.24 mg/kg/wk	I: 109 C:115	52 weeks	3
Horikawa et al.^33^	NCT03874013	12-month, open-label, multicenter, randomized, active-controlled, parallel-group, phase 3 study	Somatrogon	0.25, 0.48, and 0.66 mg/kg/wk	Daily GH	0.18 mg/kg/wk	I: 22 C:22	52 weeks	2
Zelinska et al.^34^	NCT01592500	Phase 2 safety and dose-finding study	Somatrogon	0.25, 0.48, 0.66 mg/kg/wk	Daily GH	0.24 mg/kg/wk	I: 42 C:11	52 weeks	3

*Wk* weeks.

### Publication bias assessment

Begg rank correlation test and Egger’s test showed that there was no publication bias (*p* > 0.05). The funnel plots are presented in Supplementary Fig. 1. For HV outcome, six ^18,28,30,33–35^ studies showed publication bias (*p* < 0.01). Therefore, these articles were removed and re-analysis was performed with the remaining studies to evaluate HV as an efficacy parameter in children with GHD. HV and HSDS had *p* = 0.7194 (Supplementary Fig. 1A.) and *p* = 0.0802 (Supplementary Fig. 1B.), respectively, whilst, safety showed *p* = 0.9768 (Supplementary Fig. 1C.) indicating no publication bias.

### Heterogeneity analysis of baseline characteristics

Heterogeneity analysis revealed that the baseline demographics for all the parameters such as age, basal height velocity, height SDS, bone age, body mass index and GH peak was within the normal range of 0–30% and hence comparable across all the included studies.

### Efficacy of growth hormones on height velocity

**Figure 2:**
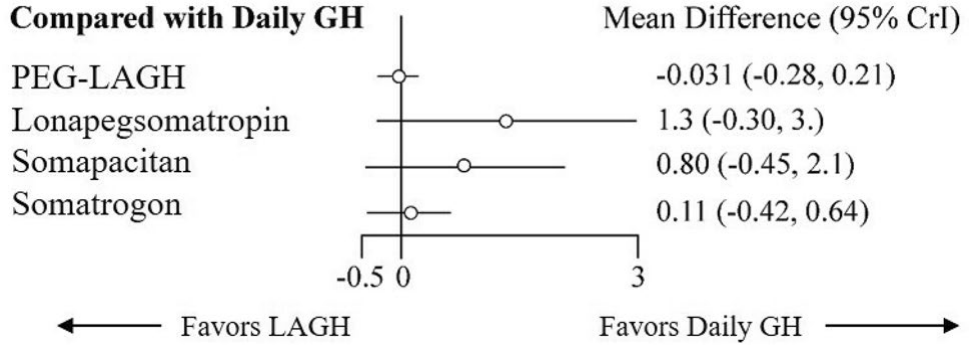
Forest plot comparing HV between LAGH and DGH. *CrI* credible interval; *GH* growth hormone; *HV* height velocity; *LAGH* long-acting growth hormone.

PEG-LAGH exhibited better effect on HV (MD: -0.031, 95% CrI: -0.278, 0.215) than somatrogon (MD: 0.105, 95% CrI: -0.419, 0.636), somapacitan (MD: 0.802, 95% CrI: -0.451, 2.068) and lonapegsomatropin (MD: 1.335, 95% CrI: -0.3, 2.989) when compared with DGH (Fig. 2). Consistent with the forest plot, PEG-LAGH ranked the highest in SUCRA (0.78), followed by DGH (0.72), somatrogon (0.61), somapacitan (0.26) and lonapegsomatropin (0.12) (Supplementary Table 2).

### Efficacy of growth hormones on HSDS

**Figure 3:**
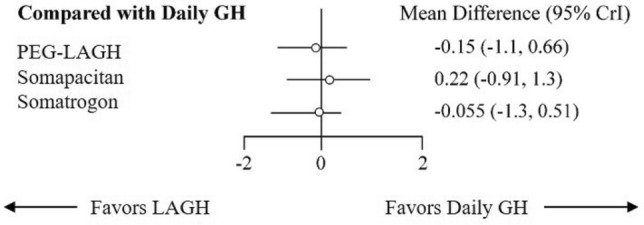
Forest plot comparing HSDS between LAGH and DGH. *CrI* credible interval; *GH* growth hormone; *HSDS* height standard deviation score; *LAGH* long-acting growth hormone.

HSDS as an outcome was evaluated in seven studies^16,18,22,31,33–35^. PEG-LAGH had better effect on HSDS (MD: -0.15, 95% CrI: -1.1, 0.66) than somatrogon (MD: -0.055, 95% CrI: -1.3, 0.51) and somapacitan (MD: 0.22, 95% CrI: -0.91, 1.3) when compared with DGH (Fig. 3). PEG-LAGH ranked the highest in terms of SUCRA (0.68), followed by somatrogon (0.56), DGH (0.49) and somapacitan (0.27) (Supplementary Table 2).

### Safety assessment

**Figure 4:**
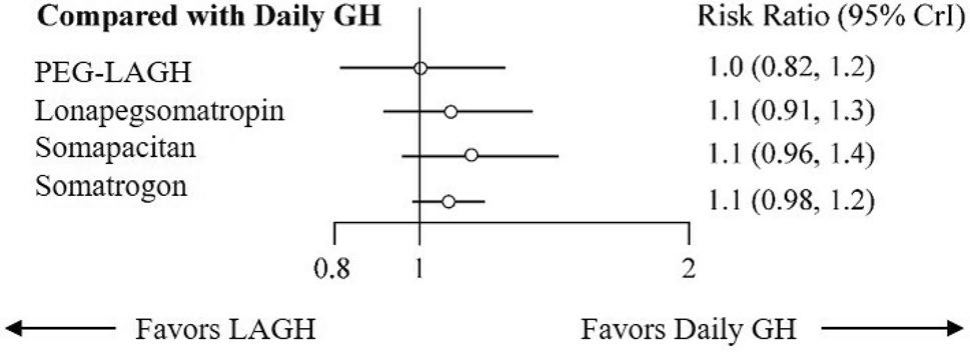
Forest plot comparing AEs between LAGH and DGH. *AEs* adverse events; *CrI* credible interval; *GH* growth hormone; *LAGH* long-acting growth hormone.

All the nine included studies reported AEs. The incidence of AEs with PEG-LAGH (RR: 1.00, 95% CrI: 0.82, 1.2) was comparable to DGH (Fig. 4). The risk of AEs with PEG-LAGH was lower compared with somatrogon (RR: 1.1, 95% CrI: 0.98, 1.2); somapacitan (RR: 1.1, 95% CrI: 0.96, 1.4) and lonapegsomatropin (RR, 1.1, 95% CrI: 0.91, 1.3). PEG-LAGH (0.68) was the second-best regimen after DGH (0.80) in terms of SUCRA, followed by lonapegsomatropin (0.41), somatrogon (0.39) and somapacitan (0.23) (Supplementary Table 2).

## Discussion

To our knowledge, this is the first study to perform a NMA of the LAGH for the treatment of GHD in prepubertal children. PEG-LAGH seems to show better efficacy in comparison with daily GH. PEG-LAGH also seemed to indicate an advantage over other LAGH in terms of HV and HSDS. In addition, PEG-LAGH had comparable and well tolerable safety profiles when compared to DGH.

The better efficacy of PEG-LAGH with respect to DGH and other LAGH as well as comparable safety to DGH and better safety to other LAGH could be attributed to differences among subjects based on ethnicity, age and auxological parameters. We thus performed a heterogeneity analysis to consider any bias in baseline characteristics from included studies. The heterogeneity was well within the normal range of 0 to 30% indicating no difference among the baseline demographics across all the included studies. Early initiation of GH replacement therapy in children with GHD helps to restore longitudinal growth^36^. Despite the efficacy, DGH injections can be burdensome for patients and their caregivers, thereby influencing the adherence and resulting in suboptimal clinical outcomes^9,37^. The advent of LAGH that require lesser number of injections are expected to ease the burden of chronic daily injections and improve adherence in patients. The results of the current study showed a better efficacy of LAGH when compared to DGH, with better HV yield observed with PEG-LAGH than somatrogon, somapacitan and lonapegsomatropin. Furthermore, PEG-LAGH had better effect on HSDS than other LAGH. In a meta-analysis by Yang et.al, no significant HV difference between high dose LAGH and DGH (MD, − 0.10; 95% CI, − 0.79 to 0.60, *p* = 0.79) was observed^20^. Similarly, another meta-analysis showed that LAGH replacement therapy had no significant difference in HV (MD, − 0.09; 95% CI, − 0.69–0.5, *p* = 0.76) when compared to DGH. However, we could not draw any comparative conclusion with regards to the improvement in final height gained by the children treated with LAGH in this NMA.

The safety of an intervention is of vital clinical importance as it could also impact the treatment decisions. In addition to the efficacy parameters, overall safety of PEG-LAGH, somatrogon, somapacitan and lonapegsomatropin were evaluated in the current study. The results of the study suggested a tolerable safety profile of LAGH when compared to DGH. This observation is in line with the previously published studies which showed the risk of total AEs and severe AEs were not significantly different in LAGH compared to controls (RR: 1.65, 95% CI: 0.83–3.29; *p* = 0.15; and RR 0.60, 95% CI: 0.30–1.19; *p* = 0.14; respectively)^19^.

The study is not without limitations. A comparatively small number of RCTs were available for the analysis. Furthermore, except for three studies, other included RCTs were of low quality as per the JADAD score. Therefore, there is a need for high-quality RCTs evaluating the efficacy and safety of LAGH with large samples. Besides, the drug dosages varied across studies which may impact data interpretation. Given that PEG-LAGH trials included only Chinese population, the baseline characteristics may influence the efficacy and safety of the findings. For this purpose, we had carried out heterogeneity analysis where all the baseline demographic characteristics were found to be comparable. Having said that, further NMAs should take into account the heterogeneity among disease characteristics, number of centers, country wise distribution and treatment duration as well to confirm the study findings. Nevertheless, this study might be helpful in guiding the physicians to choose the optimal LAGH regimen for the treatment of GHD in prepubertal children.

## Conclusion

To summarize, the indirect comparisons of the LAGH in this study may provide valuable insights for selecting an optimal LAGH for the treatment of GHD. Further studies should focus on establishing the role of LAGH in improvement of final height among children with GHD.

### Supplementary Information

Supplementary Information.

## Data Availability

The original contributions presented in the study are included in the article. Further inquiries can be directed to the corresponding author.
