# Supplementary material for: Long-acting growth hormone in the treatment of growth hormone deficiency in children: a systematic literature review and network meta-analysis
Source: Sci Rep. 2024 Apr 5;14:8061. doi: 10.1038/s41598-024-58616-4 (PMC10997584; doi:10.1038/s41598-024-58616-4)
Supplement: Supplementary file 1 — Supplementary Information. [file 41598_2024_58616_MOESM1_ESM.docx]

***Supplementary Material***

**Long-Acting Growth Hormone In The Treatment Of Growth Hormone Deficiency In Children: A Systematic Literature Review And Network Meta-Analysis**

**Jianfang Zhu^1^, Ke Yuan^1^, Sunita Rana^2^, Satya Lavanya Jakki^2^, Amit Subray Bhat^2^, Li Liang^1^ and Chunlin Wang^1*^**

*** Correspondence:** Chunlin Wang; [hzwangcl@zju.edu.cn](mailto:hzwangcl@zju.edu.cn)

**Supplementary figure 1**. Funnel plots for assessing publication bias among the studies included for A) HV, B) HSDS, and C) AEs


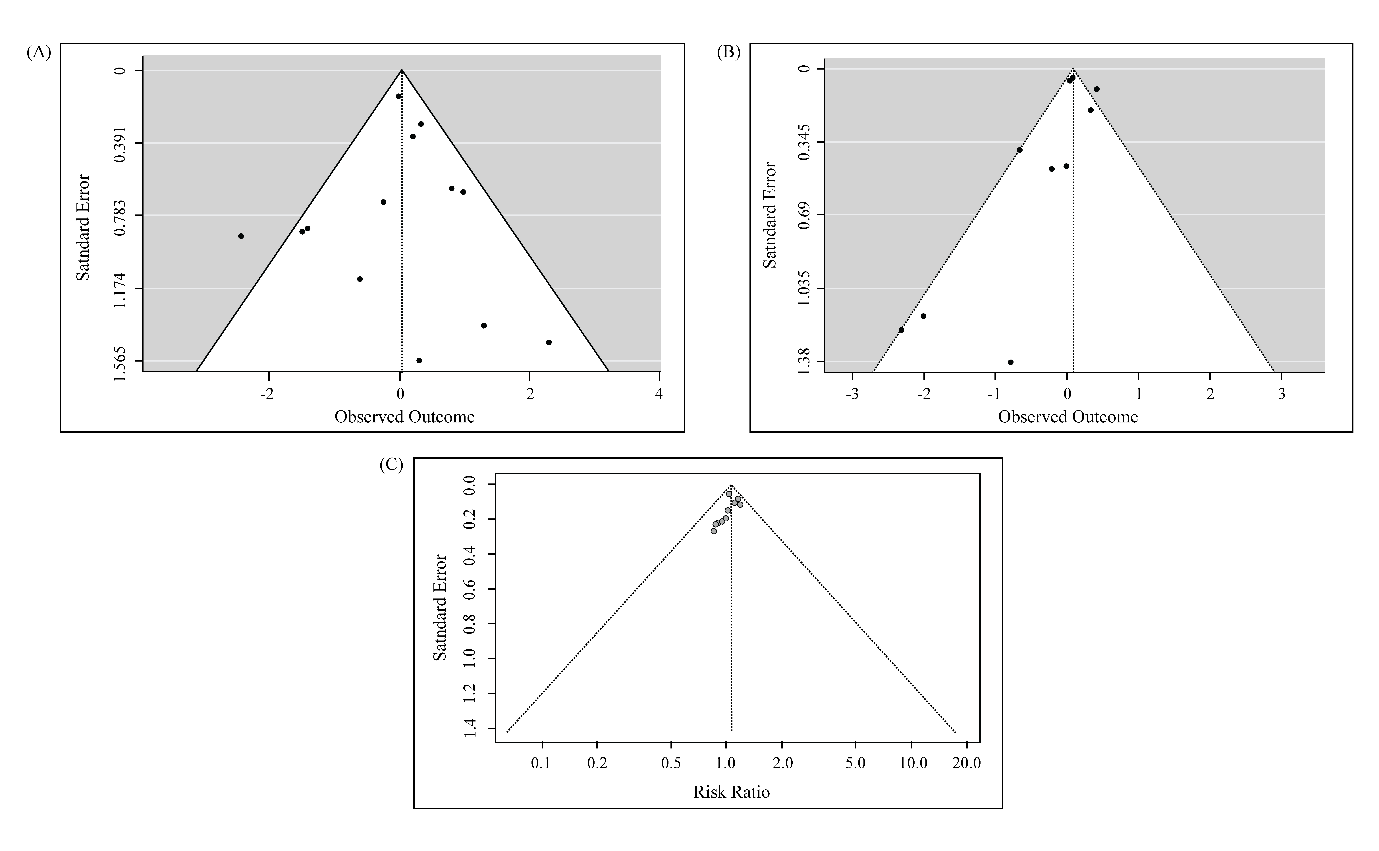


Abbreviation: AEs, adverse events; HV, height velocity; HSDS, height standard deviation score

**Supplementary Table 1**. Chinese search string

| “主题”: “Jintrolong” OR “金赛增” OR “ 聚乙二醇重组生长激素 OR “PEG-rhGh” OR “Lonapegsomatropin” OR “Skytrofa” OR “somapacitan” OR “sogroya” AND “生长激素” OR “GHD” |
| --- |

**Supplementary Table 2.** SUCRA values of treatments for efficacy and outcomes

| **Outcomes** | **Daily GH** | **PEG-LAGH** | **Somatrogon** | **Lonapegsomatropin** | **Somapacitan** |
| --- | --- | --- | --- | --- | --- |
| HV | 0.72 | 0.78 | 0.61 | 0.12 | 0.26 |
| HSDS | 0.49 | 0.68 | 0.56 | - | 0.27 |
| AEs | 0.80 | 0.68 | 0.39 | 0.41 | 0.23 |

Abbreviations: AEs, adverse events; GH, growth hormone; HV, height velocity; HSDS, height standard deviation score
